# Fulfilling the PEPFAR mandate: a more equitable use of PEPFAR resources across global health

**DOI:** 10.9745/GHSP-D-13-00137

**Published:** 2013-11-14

**Authors:** Victor K Barbiero

## Abstract

As PEPFAR moves beyond its “emergency stage,” it should now help support a more sustainable development mode, including an equitable platform for meeting a broad range of priority health needs, while continuing to pursue the goal of an AIDS-free generation.

## INTRODUCTION

It is time to implement the broader United States Government (USG) global health mandate while maintaining priority for the most effective health interventions. The political and policy mandate exists to invest in a more flexible and more equitable approach. The recent Institute of Medicine (IOM) evaluation of PEPFAR applauds PEPFAR's impressive successes.^1^ At the same time, the IOM points out 2 major challenges for PEPFAR: (1) to better address prevention through behavior change, and (2) to shift the burden and responsibility of programming more to the affected countries. Also, the President's Global Health Initiative (GHI) acknowledges the huge and compelling global health needs beyond those of HIV, including principles that focus on women, girls, and gender equity, and on health systems strengthening. The policy mandate for a broader PEPFAR approach already exists in its Hyde–Lantos authorizing legislation^2^:

- Section 4(6) (A) states: *“the USG should strengthen primary health care systems”*
- Section 301(b)(1)(B), Title III; Subtitle A states: *“It is the policy objective of the United States to strengthen the capacity to deliver primary health care in developing countries, especially in sub-Saharan Africa …”*

## THE NUMBERS SPEAK FOR THEMSELVES: TOO MANY CHILDREN DIE EACH YEAR

Approximately 6.9 million people die annually before their fifth birthday from preventable causes.^3^^,^^4^ By comparison, approximately 1.7 million people of all ages die from HIV^5^; about 1.4 million die from TB^6^; and about 655,000 die from malaria.^7^ Thus, approximately 3.76 million people of all ages die each year from HIV, TB, and malaria combined—about 10,300 per day—an extraordinary and important number, to be sure. Tragically, however, about 6.04 million children under 5 years old also die annually from causes other than HIV, TB, or malaria (approximately 16,500 per day) (Table 1).

**Table 1.** Under-5 Deaths Excluding AIDS, Tuberculosis (TB), and Malaria

**Cause of Child Deaths**	**Approximate No. of Child Deaths**
AIDS	230,000
TB	75,000
Malaria	560,000
**Total child deaths from AIDS/TB/malaria**	**865,000**
Total under-5 deaths worldwide	6,900,000
Total child deaths from AIDS/TB/malaria	(865,000)
**Annual under-5 deaths excluding AIDS/TB/malaria**	**6,035,000**

Source: References 3–7.

USG efforts can save the lives of millions more children annually through broader and more equitable use of the tremendous resources already available in global health programs, especially those within the purview of PEPFAR. Simply put, we just have to spend the money in different ways. The work of PEPFAR deserves kudos, as does the prevention and treatment efforts of the President's Malaria Initiative (PMI), investments to reduce TB mortality, and support for the Global Polio Eradication Initiative (GPEI). However, *the verticality of these programs is their Achilles' heel. A more equitable approach is required for global health programming.*

**Figure 1.**
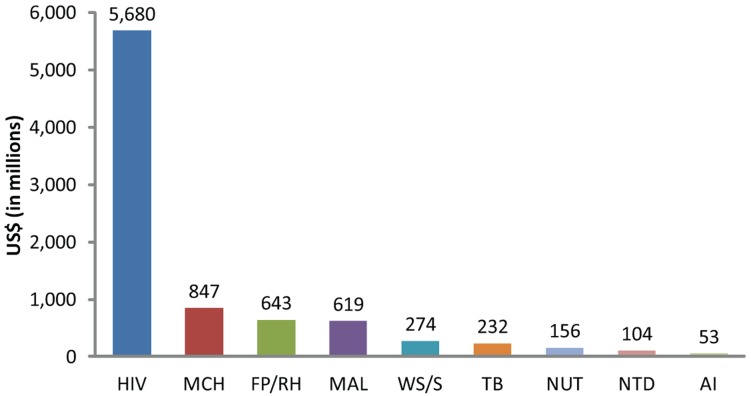
U.S. Government Health Sector Allocations, 2013 Abbreviations: AI, avian influenza; FP/RH, family planning/reproductive health; MAL, malaria; MCH, maternal and child health; NTD, neglected tropical diseases; NUT, nutrition; TB, tuberculosis; WS/S, water safety/sanitation. Source: Reference 8.

U.S. foreign assistance funding has increased in recent years, but it is grossly skewed (Figure 1 and Table 2). For example, in fiscal year 2013, the Congressional request for global health included US$5.68 billion for HIV and $847 million for maternal and child health.^8^ In fiscal year 2010, USAID/Kenya received approximately $548 million for HIV/AIDS.^9^ To put these resources in another perspective, in fiscal year 2010 the entire budget of the U.S. Peace Corps was $400 million.^10^ Such inequities detract from the efficiency and effectiveness of USG health investments worldwide and levy an opportunity cost on other preventive and curative health interventions for children and mothers.

**Table 2.** PEPFAR Kenya-Approved Funding 2010

**Agency**	**PEPFAR Funding (US$)**
Department of Defense	23,546,982
Department of Health and Human Services	166,172,628
U.S. Agency for International Development	348,654,368
Department of State	9,745,463
**TOTAL**	**548,119,441**

Source: Reference 9.

The crucial question is: *Rather than devoting the majority of our global health resources to sharply focused vertical efforts, can we implement a more flexible, more equitable resource base that will promote broader, more sustainable health development priorities while also achieving all of the (vertical) HIV, TB, malaria (and other) program objectives?* **The answer is “yes.”**

A vigorous debate is underway over vertical versus broader funding for global health assistance.^11^^,^^12^ Some talk about diagonal funding through the Global Fund to Fight AIDS, Tuberculosis and Malaria.^13^ Others contend that PEPFAR has significantly strengthened general health systems.^14^ Advocates of vertical programming fear that broader use of resources will dilute impacts and delay success. But others question the epidemiologic rationale, sustainability, absorptive capacity, accountability, and opportunity costs associated with huge vertical appropriations, particularly in light of virtually flat-lined or proportionately declining appropriations in investments that equitably reflect epidemiological priorities.^15^^–^^17^

Currently, many program objectives remain sequestered in initiative-specific silos, and the bulk of global health resources remain vertically programmed within those silos. A broader, developmental approach would strengthen PEPFAR outcomes, save the lives of millions, and promote local ownership and sustainability. PEPFAR has transitioned from an emergency program to a more development-oriented program. At the country level, many USG programs seek to achieve the specific objectives of PEPFAR, while at the same time they integrate resources to promote lower-level system strengthening for key services such as routine immunizations, well- and sick-child care, newborn care, nutrition interventions, family planning, malaria prevention, and improved referral to reduce all-cause mortality and morbidity among women and children.

## PROMOTING SYNERGY WHILE MAINTAINING PRIORITIES

A clear rationale exists for broadening PEPFAR programming: *If you strengthen elements of the primary health system (including crucial public health components) in addition to HIV/AIDS service delivery, you will increase community ownership, overall quality of services, trust in the system, and sustainability, and, ultimately, this will result in greater use of more comprehensive health services by the local population.* Thus, greater PEPFAR investments in strengthening the platforms for service delivery will increase client draw, service availability, and health system use. In turn, this will not only achieve the objectives of PEPFAR but also improve the overall health of the community. A real win-win potential exists if we work smarter with PEPFAR (and PMI, TB, GPEI, and other) resources. We can achieve PEPFAR objectives and also save more of those 6.0 million children dying from non-HIV, TB, or malaria causes.

**Figure 2.**
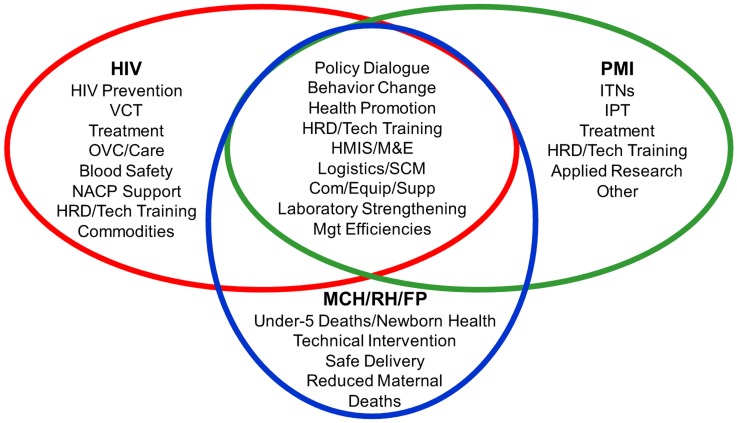
Health Systems Synergies: HIV, PMI, MCH/RH/FP Abbreviations: Com/Equip/Supp, commodities/equipment/supplies; HMIS, health management information system; HRD, human resource development; IPT, intermittent preventive therapy; ITNs, insecticide-treated bed nets; M&E, monitoring and evaluation; MCH/RH/FP, maternal and child health/reproductive health/family planning; Mgt, management; NACP, National AIDS Control Program; OVC, orphans and vulnerable children; PMI, President’s Malaria Initiative; SCM, supply chain management; Tech, technology; VCT, voluntary counseling and testing.

In many countries, particularly in Africa, U.S. foreign assistance often includes support for a wide range of health programming—HIV, TB, malaria, child survival, maternal health, reproductive health, family planning, and other infectious diseases. Figure 2 illustrates some of the potential *“systems synergies”* among HIV, PMI (and TB), and MCH/RH/FP programs. Many of the system elements that HIV, TB, and malaria efforts require to achieve their objectives also are required for saving the lives of children and mothers dying from other causes. Building on such synergies offers an evident economy of scale that is affordable, managerially efficient, technically sound, and also politically mandated by current USG policy. Although PEPFAR currently promotes some of these synergies, we need to do more.

Maximizing a more equitable distribution of PEPFAR resources will require setting specific objectives—both outcomes and impacts—for improving child and maternal health. In order to realize a broader and more equitable distribution of global health resources, PEPFAR (and all other current special initiatives—for example, PMI, TB, GPEI, Feed the Future) should include specific additional priorities for strengthened health systems, strengthened capacity to deliver primary health services, including high-impact preventive services, and improved child and maternal health. We should embrace the following in all aspects of PEPFAR programming through the end of the decade:

- In addition to pursuing the stated objectives of treatment, care, and prevention for HIV/AIDS in countries that receive PEPFAR support, PEPFAR should aim to reduce child and maternal mortality by at least 20% by the end of the decade via broader, more equitable investments in systems strengthening, including infrastructure, human resource development, and sustained service delivery. Specifically:
  - **For children:** Reduce under-5 mortality from respiratory disease, diarrheal disease, immunizable diseases, and neonatal/perinatal causes such as preterm birth, sepsis, trauma, pneumonia, tetanus, and hypothermia
  - **For mothers:** Reduce pregnancy-related deaths of women through improved family planning service delivery, appropriate management of pregnancy and labor, improved maternal nutrition, and improved girls' education

We cannot continue to allow the 6.0 million deaths of children annually from non-HIV, TB, or malaria causes. Technically, politically, and morally, we have an imperative to promote a more balanced distribution of global health resources and to maximize the impact of these resources. The policy mandate is clear for the U.S. foreign assistance architecture to enter a new era of more equitable and sustainable efforts aimed at reducing global mortality and morbidity for mothers and children under 5 while simultaneously achieving an AIDS-free generation. With an expanded agenda and specific objectives crafted to further fulfill its initial mandate, PEPFAR could accomplish much toward meeting these goals. ***–Victor K Barbiero, Associate Editor***
